# Association of High Serum Adiponectin Level With Adverse Cardiovascular Outcomes and Progression of Coronary Artery Calcification in Patients With Pre-dialysis Chronic Kidney Disease

**DOI:** 10.3389/fcvm.2021.789488

**Published:** 2022-01-13

**Authors:** Sang Heon Suh, Tae Ryom Oh, Hong Sang Choi, Chang Seong Kim, Joongyub Lee, Yun Kyu Oh, Ji Yong Jung, Kyu-Beck Lee, Kook-Hwan Oh, Seong Kwon Ma, Eun Hui Bae, Soo Wan Kim

**Affiliations:** ^1^Department of Internal Medicine, Chonnam National University Medical School and Chonnam National University Hospital, Gwangju, South Korea; ^2^Department of Prevention and Management, School of Medicine, Inha University, Incheon, South Korea; ^3^Department of Internal Medicine, Seoul National University, Seoul, South Korea; ^4^Division of Nephrology, Department of Internal Medicine, Gachon University of Gil Medical Center, Incheon, South Korea; ^5^Department of Internal Medicine, Kangbuk Samsung Hospital, Sungkyunkwan University School of Medicine, Seoul, South Korea

**Keywords:** adiponectin, cardiovascular disease, cardiovascular event, chronic kidney disease, coronary artery calcification

## Abstract

**Background:** Serum adiponectin level predicts cardiovascular (CV) outcomes and progression of coronary artery calcification (CAC) in the general population, although the association has not been validated in patients with chronic kidney disease (CKD). In this study, we investigated the association of high serum adiponectin level with the risk of adverse CV outcomes and progression of CAC in patients with pre-dialysis CKD.

**Methods:** A total of 1,127 patients with pre-dialysis CKD from a nationwide prospective cohort of patients with pre-dialysis CKD in Korea were divided into the tertile by serum adiponectin level at the baseline. CV outcome of interest was fatal and non-fatal CV events and all-cause mortality. Progression of CAC was defined as coronary artery calcium score (CACS) change more than 200 during a 4-year follow-up.

**Results:** Cox regression analysis revealed that high serum adiponectin is associated with increased risk of fatal and non-fatal CV events (adjusted hazard ratio 2.799, 95% CI 1.348–5.811). In contrast, high serum adiponectin level was not significantly associated with all-cause mortality (adjusted hazard ratio 0.655, 95% CI 0.203–2.113). Binary logistic regression analysis revealed that high serum adiponectin level is also associated with increased risk of progression of CAC (adjusted odds ratio [OR] 2.078, 95% CI 1.014–4.260). Subgroup analyses demonstrated that the association of high serum adiponectin with increased risk of fatal and non-fatal CV events is not modified by age, gender, history of diabetes, estimated glomerular filtration rate (eGFR), or spot urine albumin-to-creatinine ratio (ACR).

**Conclusions:** High serum adiponectin level is associated with adverse CV outcomes and progression of CAC in patients with pre-dialysis CKD.

## Introduction

Adiponectin is a cytokine released from adipose tissue (1). Its full-length form consists of 244 amino acids, with a molecular weight of 28 kDa, although a globular form is also generated by proteolytic cleavage (2, 3). The biological action is mediated by its major receptors, AdiopoR1 and AdipoR2 (4), which are very similar in the structures, but are distinguished by their different affinities to adiponectin and *in vivo* distributions (4, 5). Adiponectin is pleiotropically organ-protective *via* anti-oxidant and anti-inflammatory processes under physiologic conditions (6). This is well-illustrated by the stabilization of vulnerable plaques after overexpression of adiponectin in mice (7), thereby inhibiting the progression of pre-existing atherosclerotic lesions. Several studies also reported low adiponectin levels as a predictor of coronary artery calcification (CAC) progression in human subjects (8–10). Further, a pharmacologic intervention with telmisartan and statins increased serum adiponectin levels, which was associated with the reduction of cardiac events (8). It is, therefore, now widely accepted that low serum adiponectin level predicts the long-term cardiovascular (CV) outcomes in the general population (11).

It should be, however, reminded that the role of adiponectin is context-dependent. The associations of low serum adiponectin with metabolic syndrome and systolic blood pressure (SBP) were found only in the female (12) and male subjects (13), respectively, suggesting that the mode of action may be modified in a gender-dependent manner. A report from Estonia aimed to investigate gender-specific associations between metabolic syndrome and serum adiponectin level revealed that, in a fully adjusted, serum adiponectin was significantly associated with metabolic only in women (12). A study targeting the association of adipokines with blood pressure in rural Chinese adolescents reported that adiponectin was negatively associated with SBP only in men (13). Mounting evidence also suggests that chronic kidney disease (CKD) should alter the clinical interpretation of serum adiponectin level, as low serum adiponectin is associated with better health-related quality of life in a cohort study of patients with pre-dialysis CKD (14). Another study from the same cohort also reported the increased risk of anemia development with high serum adiponectin (15). A study from Taiwan with a mean follow-up duration of 5 years reported that high serum adiponectin increased the risk of end-stage renal disease in patients with non-diabetic CKD (16). These all together suggest that the role of adiponectin could be considerably altered in patients with CKD, which could be primarily attributed to alterations in the clearance of circulating adiponectin (17). Yet, it has not been elucidated whether the conventional association between adiponectin and CAC is still valid in patients with CKD.

We here investigated the association of high serum adiponectin level with CV outcomes in patients with pre-dialysis CKD. We also examined the association of serum adiponectin level with the progression of CAC, as coronary artery disease (CAD) is the leading cause of mortality in patients with CKD (18).

## Methods

### Study Designs and Participants

**Figure 1:**
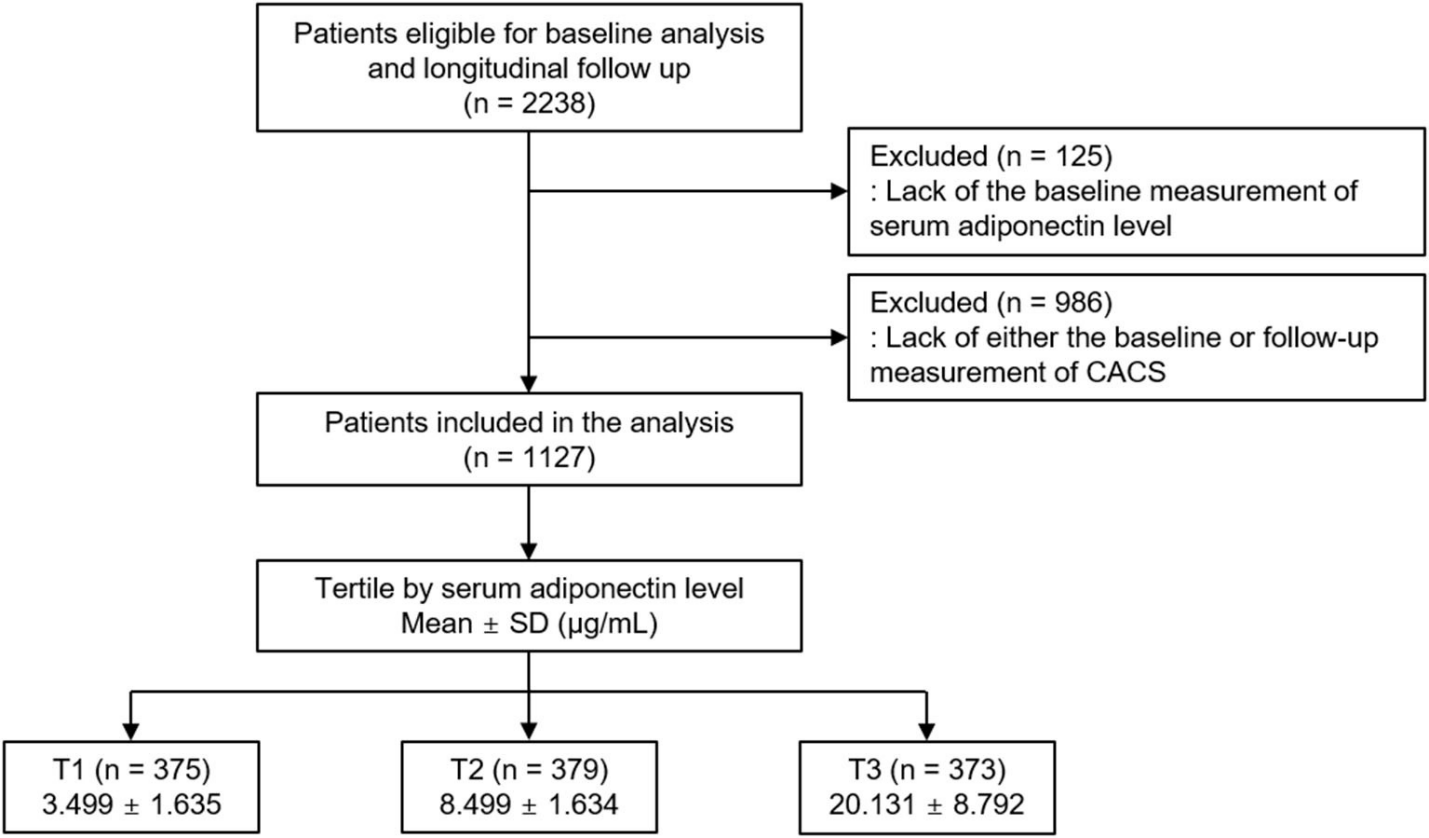
Flow diagram of the study participants. CACS, coronary artery calcium score; SD, standard deviation; CACS, coronary artery calcium score; T1, 1st tertile; T2, 2nd tertile; T3, 3rd tertile.

The Korean Cohort Study for Outcomes in Patients With Chronic Kidney Disease (KNOW-CKD) is a nationwide prospective cohort study involving 9 tertiary-care general hospitals in Korea (NCT01630486 at http://www.clinicaltrials.gov) (19). Korean patients with CKD from stage 1 to pre-dialysis stage 5, who voluntarily provided informed consent, were enrolled between 2011 and 2015. All participants had been under close observation, and participants who experienced study outcomes were reported by each participating center. The study observation period ended on March 31, 2020. A total of 2,238 subjects were longitudinally followed up (Figure 1). After excluding those lacking the baseline measurement of serum adiponectin level, and those lacking either the baseline or follow-up measurement of coronary artery calcium score (CACS), 1,127 subjects were finally included for the analyses. The median follow-up duration was 6.962 years.

### Data Collection

Demographic information was collected from all eligible participants, including age, gender, smoking history, medications (angiotensin-converting enzyme inhibitors and angiotensin receptor blockers (ACEi/ARBs), diuretics, and statins), and comorbid conditions, at the time of screening. Anthropometric indices [height, weight circumference (WC), SBP, and diastolic blood pressure (DBP)] were also measured. Body mass index (BMI) was calculated as weight/height^2^ (kg/m^2^). Laboratory data included hemoglobin, creatinine, albumin, glucose, triglyceride, total cholesterol, low-density lipoprotein cholesterol, high-density lipoprotein cholesterol (HDL-C), and high sensitive C-reactive protein (hs-CRP). Serum creatinine was measured by an isotope dilution mass spectrometry–traceable method, and estimated glomerular filtration rate (eGFR) was calculated by Chronic Kidney Disease Epidemiology Collaboration (CKD-EPI) equation (20). CKD stages were determined by the Kidney Disease Improving Global Outcomes guidelines (12). For example, the subjects with persistent albuminuria [spot urine albumin-to-creatinine ratio (ACR) ≥ 30 mg/gCr] for more than 3 months and eGFR >90 ml/min/1.73 m^2^ were classified into stage 1. Urine ACR was measured in random, preferably first-voided, spot urine samples. The 24 h urine protein excretion was also determined.

### Determination of Serum Adiponectin Level

Serum adiponectin level was measured using a commercial enzyme-linked immunosorbent assay kit (Adipogen Corp., San Diego, CA, USA). This method had intra- and inter-assay coefficients of variations of ≤ 3.8 and ≤ 5.5%, respectively. The subjects were divided into the tertile (T1–T3) by serum adiponectin levels (Figure 1). The cut-offs at T1 and T3 were <6.041 μg/ml and ≥11.884 μg/ml, respectively. T3 was defined as high serum adiponectin level.

### Study Outcomes

The outcomes of interest were fatal and non-fatal CV events and all-cause mortality. CV events, either fatal or non-fatal, included any coronary artery event (unstable angina, myocardial infarction, or coronary intervention/surgery), hospitalization for heart failure, ischemic or hemorrhagic stroke, incident peripheral arterial disease, and symptomatic arrhythmia. Progression of CAC was defined as an increase in CACS of more than 200 AUs during a 4-year follow-up (21).

### Statistical Analysis

Continuous variables were expressed as mean ± SD or median [interquartile range]. Categorical variables were expressed as a number of participants and percentage. For descriptive analyses, Student's t-test or one-way ANOVA and χ^2^ test were used for continuous and categorical variables, respectively. In the primary analysis, the participants with any missing data were excluded for further analyses. To assess the association between high serum adiponectin level and the outcomes, Cox proportional hazard regression models were analyzed. Patients lost to follow-up were censored at the date of the last visit. We adjusted age, gender, Charlson comorbidity index, history of diabetes mellitus (DM), smoking history, BMI, WC, SBP, and DBP, medication (ACEi/ARBs, diuretics, and statins), hemoglobin, albumin, HDL-C, fasting glucose, hs-CRP, eGFR, 24-h urine protein, and categorized CACS at the baseline. The results of Cox proportional hazard models were presented as hazard ratios (HRs) and 95% CIs. Restricted cubic splines were used to visualize the association between serum adiponectin as a continuous variable and the HR for fatal and non-fatal CV events or all-cause mortality. To address the association between high serum adiponectin level and progression of CAC, binary logistic regression models were analyzed. As progression of CAC in subjects with CACS 0 AU at the baseline was extremely rare (Supplementary Figure S1), those with baseline CACS 0 AU were excluded from the analyses (n = 605). The model was also adjusted for age, gender, Charlson comorbidity index, history of DM, smoking history, BMI, WC, SBP, and DBP, medication (ACEi/ARBs, diuretics, and statins), hemoglobin, albumin, HDL-C, fasting glucose, hs-CRP, eGFR, 24-h urine protein, and categorized CACS at the baseline. The results of binary logistic regression models were presented as odds ratios (ORs) and 95% CIs. To substantiate our findings, we performed sensitivity analyses by excluding the subjects with eGFR ≥ 90 ml/min/1.73 m^2^, since the subjects with eGFR ≥ 90 ml/min/1.73 m^2^ were considered close to normal kidney function. In addition, we replaced missing values in primary analyses with multiple imputation and further conducted Cox regression and binary logistic regression analyses. Two-sided *p* < 0.05 were considered statistically significant. Statistical analysis was performed using SPSS for Windows version 22.0 (IBM Corp., Armonk, NY, USA) and R (version 3.4.3; www.r-project.org; R Foundation for Statistical Computing, Vienna).

## Results

### Baseline Characteristics

The baseline characteristics of study participants were described by serum adiponectin level (Table 1). The frequency of male gender, history of DM, and current smokers was highest in T1. The other demographic data revealed no significant difference among the groups. BMI and WC were also highest in T1, while waist-to-hip ratio, SBP, and DBP were not significantly different across the groups. Hemoglobin and albumin levels were highest in T1. HDL-C level was highest in T3, while triglyceride level was highest in T1. Fasting glucose and hs-CRP levels were highest in T1. Interestingly, spot urine ACR and eGFR were lowest in T1 and T3, respectively. Collectively, low serum adiponectin level was largely associated with unfavorable clinical features at the baseline, whereas its serum level was inversely correlated with renal function.

**Table 1:** Baseline characteristics of study participants in the tertile by serum adiponectin level.

	**Missing (%)**	**Adiponectin**			
		**T1**	**T2**	**T3**	***P* value**
CACS (AU)	0.0				0.057
0		187 (49.9)	192 (50.7)	226 (60.6)	
0 <, ≤400		161 (42.9)	159 (42.0)	124 (33.2)	
400 <, ≤1,000		19 (5.1)	416 (4.2)	16 (4.3)	
1000 <		8 (2.1)	12 (3.2)	7 (1.9)	
Age (year)	0.0	51.0421 ± 11.574	53.095 ± 11.996	52.828 ± 11.975	0.115
Male	0.0	283 (75.5)	214 (56.5)	163 (43.7)	< 0.001
Charlson comorbidity index	0.0				0.898
0–3		308 (82.1)	311 (82.1)	306 (82.0)	
4–5		65 (17.3)	66 (17.4)	83 (16.9)	
≥ 6		2 (0.5)	2 (0.5)	4 (1.1)	
DM	0.0	118 (31.5)	79 (21.2)	93 (24.5)	0.005
CAD	0.0	6 (1.6)	10 (2.6)	5 (1.3)	0.378
Arrhythmia	0.0	6 (1.6)	2 (0.5)	7 (1.9)	0.233
Current smoking	0.0	75 (20.0)	59 (15.6)	41 (11.0)	0.003
Medication	0.0				
ACEi/ARBs	0.0	333 (88.8)	327 (86.3)	313 (83.9)	0.151
Statins	0.0	193 (51.5)	200 (52.8)	175 (46.9)	0.243
Diuretics	0.0	90 (24.0)	101 (26.6)	91 (24.4)	0.663
BMI (kg/m^2^)	0.3	25.377 ± 3.076	24.820 ± 3.525	23.606 ± 3.213	< 0.001
WC (cm)	6.1	89.677 ± 8.610	87.603 ± 9.307	84.322 ± 10.103	< 0.001
WHR	6.1	0.908 ± 0.056	0.897 ± 0.061	0.880 ± 0.074	< 0.001
SBP (mmHg)	0.0	126.691 ± 14.311	125.573 ± 14.396	125.429 ± 15.183	0.434
DBP (mmHg)	0.0	77.181 ± 10.076	76.322 ± 10.214	77.147 ± 10.970	0.440
Laboratory findings					
Hemoglobin (g/dL)	1.3	14.041 ± 1.872	13.301 ± 1.737	12.724 ±1.648	< 0.001
Albumin (g/dL)	0.5	4.346 ± 0.307	4.247 ± 0.346	4.194 ± 0.357	< 0.001
Total cholesterol (mg/dL)	0.0	171.363 ± 36.720	174.158 ± 38.523	176.386 ± 39.632	0.199
HDL-C (mg/dL)	1.4	45.814 ± 12.507	50.477 ± 13.863	56.537 ± 16.700	< 0.001
LDL-C (mg/dL)	0.0	95.918 ± 31.648	95.792 ± 31.539	97.332 ± 31.583	0.760
Triglyceride (mg/dL)	2.5	176.945 ± 110.082	157.643 ± 100.184	122.036 ± 61.395	< 0.001
Fasting glucose (mg/dL)	1.0	111.086 ± 33.582	106.931 ± 29.716	101.973 ± 274.356	< 0.001
hsCRP (mg/dL)	6.6	0.900 [0.323, 1.818]	0.600 [0.200, 1.500]	0.400 [0.123, 1.153]	0.025
Spot urine ACR (mg/g Cr)	1.9	188.359 [32.569, 188.359]	263.328 [59.798, 692.853]	263.037 [36.621, 698.553]	0.015
24-hr urine protein (mg/dL)	11.8	394.000 [129.900, 965.725]	424.200 [132.000, 976.000]	372.000 [139.975, 1,017.900]	0.326
eGFR (mL/min./1.73 m^2^)	0.0	65.609 ± 29.601	57.878 ± 28.112	54.580 ± 28.558	< 0.001
CKD stages	0.0				< 0.001
Stage 1		101 (26.9)	68 (18.2)	75 (19.8)	
Stage 2		111 (29.6)	75 (20.1)	95 (25.3)	
Stage 3a		65 (17.3)	81 (21.7)	73 (19.3)	
Stage 3b		72 (19.2)	88 (23.6)	91 (24.0)	
Stage 4		25 (6.)	56 (15.0)	42 (11.1)	
Stage 5		1 (0.3)	5 (1.3)	2 (0.5)	

*Values for categorical variables are given as a number (percentage); values for continuous variables, as mean ± SD or median [interquartile range]*.

*ACEi, angiotensin-converting enzyme inhibitor; ACR, albumin-to-creatinine ratio; AU, Agatston unit; ARB, angiotensin receptor blocker; BMI, body mass index; CAD, coronary artery disease; CKD, chronic kidney disease; DBP, diastolic blood pressure; DM, diabetes mellitus; eGFR, estimated glomerular filtration rate; HDL-C, high density lipoprotein cholesterol; hsCRP, high-sensitivity C-reactive protein; LDL-C, Low density lipoprotein cholesterol; SBP, systolic blood pressure; T1, 1st tertile; T2, 2nd tertile; T3, 3rd tertile; WC, waist circumference; WHR, Waist-to-hip ratio*.

### Association of High Serum Adiponectin Level With Adverse CV Outcomes in Patients With Pre-dialysis CKD

To define the association of serum adiponectin level with clinical outcomes, Cox regression models were analyzed (Table 2). Cox regression analyses revealed that high serum adiponectin is associated with an increased risk of fatal and non-fatal CV events (adjusted HR 2.799, 95% CI 1.348–5.811, *p* = 0.006). In contrast, high serum adiponectin level was not significantly associated with all-cause mortality (adjusted HR 0.655, 95% CI 0.203–2.113, *p* = 0.479).

**Table 2:** Cox regression analysis of serum adiponectin levels for clinical outcomes.

		**Number of events (%)**	**Unadjusted HR (95%CIs)**	***P* value**	**Adjusted HR (95%CIs)**	***P* value**
Fatal and non-fatal CV events	Adiponectin, T1	20 (5.3)	1.195 (0.545, 2.619)	0.657	1.283 (0.558, 2.948)	0.558
	Adiponectin, T2	18 (4.7)	Reference		Reference	
	Adiponectin, T3	31 (8.3)	2.276 (1.153, 4.492)	0.018	2.799 (1.348, 5.811)	0.006
All-cause mortality	Adiponectin, T1	6 (1.6)	0.745 (0.243, 2.277)	0.605	0.994 (0.282, 3.499)	0.992
	Adiponectin, T2	9 (2.4)	Reference		Reference	
	Adiponectin, T3	9 (2.4)	0.731 (0.254, 2.106)	0.561	0.655 (0.203, 2.113)	0.479

*Event cases are given as a number (percentage)*.

*Models were adjusted for age, gender, Charlson comorbidity index, history of DM, smoking history, BMI, WC, SBP, DBP, medications (ACEi/ARBs, statins, diuretics), hemoglobin, albumin, HDL-C, fasting glucose, hs-CRP, eGFR, 24-h urine protein, and categorized CACS at the baseline*.

*CI, confidence interval; CACS, coronary artery calcium score; eGFR, estimated glomerular filtration rate; CV, cardiovascular; HR, hazard ratio; T1, 1st tertile; T2, 2nd tertile; T3, 3rd tertile*.

**Figure 2:**
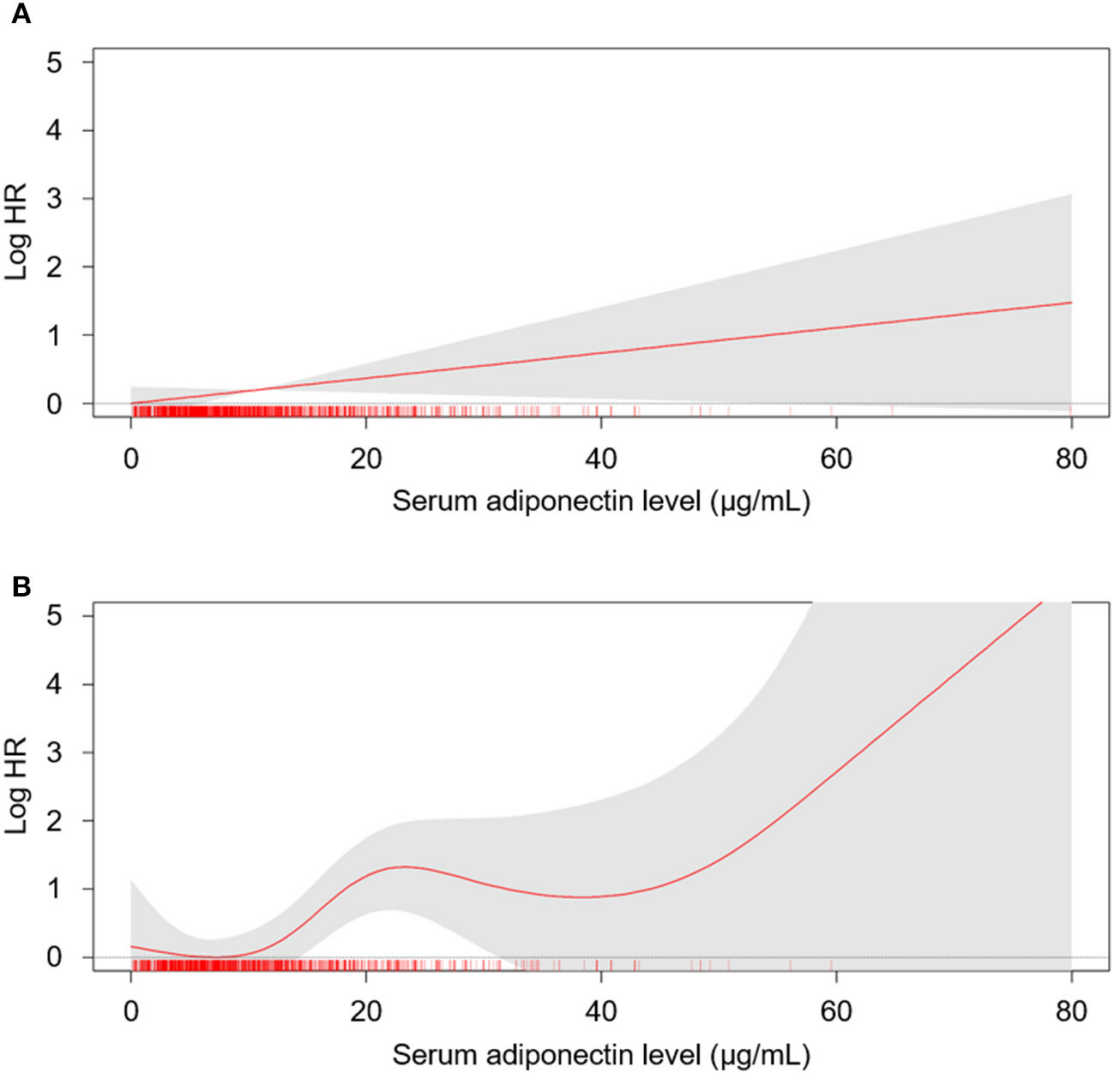
The restricted cubic spline of serum adiponectin on the risk of fatal and non-fatal CV events. Crude **(A)** and adjusted **(B)** HRs of serum adiponectin as a continuous variable for fatal and non-fatal CV events are depicted. The model was adjusted for age, gender, Charlson comorbidity index, history of DM, smoking history, BMI, WC, SBP, DBP, medications (ACEi/ARBs, statins, diuretics), hemoglobin, albumin, HDL-C, fasting glucose, hs-CRP, eGFR, 24-h urine protein, and categorized CACS at the baseline. HR, hazard ratio; CV, cardiovascular; CACS, coronary artery calcium score eGFR, estimated glomerular filtration rate; DM, diabetes mellitus; BMI, body mass index; WC, weight circumference; SBP, systolic blood pressure; DBP, diastolic blood pressure; HDL, high density lipoprotein.

To examine the linear relation of serum adiponectin level with the risk of CV events (Figure 2) and all-cause mortality (Supplementary Figure S2), we conducted spline regression analyses. Restricted cubic spine depicted that both crude (Figure 2A) and adjusted (Figure 2B) HRs for fatal and non-fatal CV events are positively correlated with serum adiponectin level. The risk of all-cause mortality was linearly correlated with serum adiponectin level in the crude model (Supplementary Figure S2A), no clear correlation was demonstrated in the adjusted model (Supplementary Figure S2B).

### Association of High Serum Adiponectin Level With the Progression of CAC in Patients With Pre-dialysis CKD

As the relation between serum adiponectin level and CV outcomes was inversed compared to the finding from the general population (8, 11), we hypothesized that high serum adiponectin level may be associated with progression, rather than stabilization, of CAC in patients with pre-dialysis CKD. The analysis of the binary logistic regression model (Table 3) revealed that high serum adiponectin level is marginally but significantly associated with increased risk of progression of CAC in patients with pre-dialysis CKD (adjusted OR 2.078, 95% CI 1.014–4.260, *p* = 0.046).

**Table 3:** Binary logistic regression of serum adiponectin levels for progression of CAC.

	**Number of events (%)**	**Unadjusted HR (95%CIs)**	***P* value**	**Adjusted HR (95%CIs)**	***P* value**
Adiponectin, T1	44 (11.7)	1.045 (0.618, 1.766)	0.869	0.978 (0.474, 2.017)	0.951
Adiponectin, T2	41 (10.8)	Reference		Reference	
Adiponectin, T3	40 (10.7)	1.328 (0.776, 2.273)	0.301	2.078 (1.014, 4.260)	0.046

*Event cases are given as a number (percentage)*.

*Models were adjusted for age, gender, Charlson comorbidity index, history of DM, smoking history, BMI, WC, SBP, DBP, medications (ACEi/ARBs, statins, diuretics), hemoglobin, albumin, HDL-C, fasting glucose, hs-CRP, eGFR, 24-h urine protein, and categorized CACS at the baseline*.

*CI, confidence interval; CACS, coronary artery calcium score; eGFR, estimated glomerular filtration rate; OR, odds ratio; T1, 1st tertile; T2, 2nd tertile; T3, 3rd tertile*.

### Sensitivity Analysis

We excluded the subjects with eGFR ≥ 90 ml/min/1.73 m^2^ who are considered close to normal kidney function (n = 244). Despite the substantial reduction of the number of subjects being analyzed, binary logistic regression analysis revealed that high serum adiponectin level is significantly associated with the progression of CAC (adjusted OR 2.179, 95% CI 1.038–4.573, *p* = 0.040; Supplementary Table S1). Cox regression analysis also demonstrated that high serum adiponectin level is significantly associated with fatal and non-fatal CV events (adjusted HR 3.096, 95% CI 1.450–6.609, *p* = 0.003), but is not significantly associated with all-cause mortality (adjusted HR 0.681, 95% CI 0.213–2.177, *p* = 0.517), even after exclusion of the subjects with eGFR ≥ 90 ml/min/1.73 m^2^ (Supplementary Table S2). In addition, we performed multiple imputation to replace the missing data in the primary analyses. High serum adiponectin was robustly associated with increased risk of fatal and non-fatal CV events in Cox regression analyses of all subjects (adjusted HR 2.099, 95% CI 1.127–3.908, *p* = 0.019) and the subjects with eGFR <90 ml/min/1.73 m^2^ (adjusted HR 1.950, 95% CI 1.019–3.733, *p* = 0.044; Supplementary Table S3). High serum adiponectin was associated with the progression of CAC in binary logistic regression analysis of the subjects with eGFR <90 ml/min/1.73 m^2^ (adjusted OR 2.055, 95% CI 1.026–4.117, *p* = 0.044), but not in the analysis of all subjects (adjusted OR 1.793, 95% CI 0.928–3.465, *p* = 0.082; Supplementary Table S4).

### Subgroup Analysis

To evaluate whether the association of high serum adiponectin level with fatal and non-fatal CV events and progression of CAC is modified by subgroups, we conducted subgroup analyses. The subgroups were stratified by age (<60 or ≥60 years), gender (male or female), history of DM (without or with), eGFR (≥ 60 or <60 ml/min/1.73 m^2^), and spot urine ACR (<300 or ≥300 mg/g). Cox regression analysis revealed that *p-*values for interactions were >0.05 for all subgroups (Table 4), suggesting that the association of high serum adiponectin with increased risk of fatal and non-fatal CV events is not modified by these factors. Binary logistic regression analysis revealed that *p*-values for interactions were >0.05 for the subgroups by gender, history of DM, eGFR, and spot urine ACR, while a marginal but significant interaction existed between age and the risk of progression of CAC (*p* for interaction = 0.047), where a non-significant trend between low serum adiponectin level and progression of CAC was observed in the subjects with age <60 years (Supplementary Table S5).

**Table 4:** Cox regression analysis of serum adiponectin levels for fatal and non-fatal CV events in various subgroups.

		**Number of events (%)**	**Unadjusted HR (95%CIs)**	***P* for interaction**	**Adjusted HR (95%CIs)**	***P* for interaction**
Age <60 years	Adiponectin, T1	11 (4.0)	1.779 (0.596, 5.310)	0.460	2.491 (0.743, 8.356)	0.375
	Adiponectin, T2	7 (2.8)	Reference		Reference	
	Adiponectin, T3	17 (6.6)	3.194 (1.170, 8.720)		3.937 (1.262, 12.278)	
Age ≥ 60 years	Adiponectin, T1	9 (9.2)	0.848 (0.248, 2.900)		0.984 (0.236, 4.108)	
	Adiponectin, T2	11 (8.7)	Reference		Reference	
	Adiponectin, T3	14 (12.2)	1.650 (0.640, 4.258)		2.879 (0.895, 9.261)	
Male	Adiponectin, T1	17 (6.0)	1.289 (0.499, 3.326)	0.876	1.352 (0.493, 3.704)	0.629
	Adiponectin, T2	11 (5.1)	Reference		Reference	
	Adiponectin, T3	17 (10.4)	2.594 (1.057, 6.364)		3.649 (1.392, 9.566)	
Female	Adiponectin, T1	3 (3.3)	0.767 (0.149, 3.955)		0.351 (0.042, 2.910)	
	Adiponectin, T2	7 (4.2)	Reference		Reference	
	Adiponectin, T3	14 (6.7)	2.071 (0.729, 5.880)		1.820 (0.442, 7.497)	
DM (-)	Adiponectin, T1	10 (3.9)	1.449 (0.442, 4.750)	0.781	2.054 (0.562, 7.513)	0.662
	Adiponectin, T2	9 (3.1)	Reference		Reference	
	Adiponectin, T3	20 (6.8)	3.332 (1.229, 9.033)		3.591 (1.226, 10.519)	
DM (+)	Adiponectin, T1	10 (8.5)	0.857 (0.300, 2.44)		1.058 (0.325, 3.445)	
	Adiponectin, T2	9 (9.7)	Reference		Reference	
	Adiponectin, T3	11 (13.9)	1.618 (0.615, 4.259)		2.252 (0.689, 7.357)	
eGFR ≥ 60 mL/min./1.73m^2^	Adiponectin, T1	9 (4.7)	1.150 (0.365, 3.628)	0.832	1.146 (0.286, 4.592)	0.636
	Adiponectin, T2	6 (4.2)	Reference		Reference	
	Adiponectin, T3	7 (5.6)	1.438 (0.439, 4.712)		1.555 (0.367, 6.596)	
eGFR <60 mL/min./1.73m^2^	Adiponectin, T1	11 (6.0)	1.198 (0.402, 3.565)		1.739 (0.540, 5.592)	
	Adiponectin, T2	12 (5.1)	Reference		Reference	
	Adiponectin, T3	24 (9.7)	2.753 (1.170, 6.478)		3.654 (1.427, 9.357)	
Spot urine ACR <300 mg/g	Adiponectin, T1	12 (5.4)	0.863 (0.313, 2.381)	0.982	0.832 (0.278, 2.491)	0.552
	Adiponectin, T2	10 (4.9)	Reference		Reference	
	Adiponectin, T3	16 (8.2)	1.822 (0.764, 4.343)		2.082 (0.797, 5.438)	
Spot urine ACR ≥ 300 mg/g	Adiponectin, T1	8 (5.4)	1.930 (0.544, 6.843)		2.054 (0.437, 9.647)	
	Adiponectin, T2	8 (4.8)	Reference		Reference	
	Adiponectin, T3	15 (8.8)	3.207 (1.049, 9.837)		9.730 (2.064, 45.860)	

*Models were adjusted for age, gender, Charlson comorbidity index, history of DM, smoking history, BMI, WC, SBP, DBP, medications (ACEi/ARBs, statins, diuretics), hemoglobin, albumin, HDL-C, fasting glucose, hs-CRP, eGFR, 24-h urine protein, and categorized CACS at the baseline*.

*ACR, albumin-to-creatinine ratio; CI, confidence interval; DM, diabetes mellitus; eGFR, estimated glomerular filtration rate; HR, hazard ratio; CV, cardiovascular; CACS, coronary artery calcium score; T1, 1st tertile; T2, 2nd tertile; T3, 3rd tertile*.

As the subgroup analysis by eGFR ≥60 or <60 ml/min/1.73 m^2^ did show any significant differences in the association between serum adiponectin level and the risk of fatal and non-fatal CV events, we tried to more sophisticatedly define the cut-off level at which eGFR level the association of serum adiponectin level and the risk of fatal and non-fatal CV events is altered (Supplementary Figure S3). In the analysis of the subjects with eGFR with ≥ 72 ml/min/1.73 m^2^ (Supplementary Figure S3A), the restricted cubic spline visualized the similar pattern shown in the analysis of the entire subjects. In contrast, in the analysis of the subjects with eGFR with ≥ 73 ml/min/1.73 m^2^ (Supplementary Figure S3B), the restricted cubic spline demonstrated a dramatically contrasting result, with an inverse correlation between serum adiponectin level and the risk of fatal and non-fatal CV events, which is similar to the pattern shown in the general population. These collectively suggest that the association of serum adiponectin and the risk of CV events in patients with CKD is altered at eGFR 70–75 ml/min/1.73 m^2^.

## Discussion

In the present study, we found that high serum adiponectin level is associated with adverse CV outcomes in patients with pre-dialysis CKD. We also discovered that high serum adiponectin level is associated with an increased risk of progression of CAC in a patient with CKD. The association of high serum adiponectin with increased risk of fatal and non-fatal CV events was not modified by age, gender, history of diabetes, eGFR, or spot urine ACR.

As CV disease is a leading cause of death in patients with CKD (22), the early detection of patients with a high risk of CV events should be an issue of particular importance in this population. Our finding in the current study is contrary to the previous reports from the general population with CAD (8–10), where low serum adiponectin level has been suggested as a predictor of CAC. The results in this study are rather in accordance with previous studies (14–16) that demonstrated the adiponectin paradox. A fundamental question to the adiponectin paradox is whether the biological action of adiponectin is actually altered in a context-dependent manner. Despite the lack of any definitive evidence from clinical trials, we speculate that organ-protective adiponectin-driven signals may be minimally or only partly affected even in clinical contexts associated with the adiponectin paradox. Indeed, overexpression of adiponectin attenuates kidney damage in a murine model of hypertensive nephropathy (23), whereas deletion of adiponectin exacerbated albuminuria and renal fibrosis in a mouse model of subtotal nephrectomy that mimics CKD in human subjects (24).

Rather, adiponectin paradox in patients with CKD could be attributed to alterations in the clearance of circulating adiponectin, as the renal clearance of adiponectin decreased in a mouse after subtotal nephrectomy, with an elevation of serum adiponectin levels (17). We also reported that despite the association of most unfavorable clinical features with low serum adiponectin level at the baseline, mean eGFR was lowest in subjects with high serum adiponectin levels (Table 1). Moreover, we found that the association of serum adiponectin and the risk of CV events is dramatically altered at eGFR 70–75 ml/min/1.73 m^2^ (Supplementary Figure S3), suggesting that adiponectin paradox in patients with CKD is already present even in early stages. Meanwhile, impaired kidney function may facilitate the accumulation of epicardial adipose tissue (EAT) (25, 26). Although EAT is a source of adiponectin (27), it also secrets detrimental adipokines, such as leptin, to promote inflammation in adjacent coronary vascular beds, the accumulation of EAT has been associated with CAD severities (6, 27). Therefore, based on the current understanding of adiponectin biology, it seems likely that the association of high serum adiponectin level with adverse CV outcomes indicates deteriorating kidney function in the natural course of CKD.

A finding of interest is that the risk of adverse CV outcomes and progression of CAS were lowest in T2, rather than in T1, although the difference between T1 and T2 was not statistically significant. One may expect that the nadir point of serum adiponectin level for adverse CV outcomes should be positioned in T3 if the analysis included the general population, as the risk of CV events inversely correlates with serum adiponectin level in the general population (8). Provided that we included patients with CKD for the analysis in which condition of the clearance of serum adiponectin is affected, it is speculated that the nadir point of serum adiponectin for the risk of adverse CV outcomes in the current study is modified along with the alteration in the overall association between serum adiponectin level and the risk of CV events. In this regard, we demonstrated that the association of high serum adiponectin level with adverse CV outcomes is valid among the subjects with only mild impairment of kidney function (Supplementary Tables S1, S2). It should be further elucidated that at which cut-off point of kidney function critically discriminates the association of serum adiponectin and CV outcomes between the general population and the patients with CKD.

## Limitations

There are a number of limitations to this study. First, we are not able to clarify the causal relationship between high serum adiponectin and adverse CV outcomes or progression of CAC, because of the observational nature of the current study. Second, although high serum adiponectin level was significantly associated with adverse CV outcomes, we could not determine the association between serum adiponectin level and all-cause mortality, because the frequency of death events was as low as 24 out of 1,127 subjects (2.7%). Third, compared to the association between high serum adiponectin level and CV outcomes, the association of high serum adiponectin level with the risk of progression of CAC is relatively marginal, although the association is repeatedly reproduced in the sensitivity analyses. Fourth, as this cohort study enrolled only ethnic Koreans, a precaution is required to extrapolate the data in the present study to other populations.

## Conclusion

In conclusion, we report that high serum adiponectin level is associated with adverse CV outcomes in patients with pre-dialysis CKD. High serum adiponectin level is also associated with an increased risk of progression of CAC in a patient with CKD. The association of high serum adiponectin with increased risk of fatal and non-fatal CV events is not modified by age, gender, history of diabetes, eGFR, or spot urine ACR.

## Data Availability Statement

The raw data supporting the conclusions of this article will be made available by the authors, without undue reservation.

## Ethics Statement

The study was conducted in accordance with the principles of the Declaration of Helsinki, and the study protocol was approved by the Institutional Review Boards of participating centers, including at Seoul National University Hospital, Yonsei University Severance Hospital, Kangbuk Samsung Medical Center, Seoul St.Mary's Hospital, Gil Hospital, Eulji General Hospital, Chonnam National University Hospital, and Pusan Paik Hospital. The patients/participants provided their written informed consent to participate in this study.

## Author Contributions

SS designed and helped in the data analysis and manuscript writing. SS, TO, and HC contributed to the conception of the study. SS and CK performed the data analyses and wrote the manuscript. YL, YO, JJ, K-BL, K-HO, and SM collected the data. EB and SK helped perform the analysis with constructive discussions. All authors contributed to the article and approved the submitted version.

## Funding

This work was supported by the Research Program funded by the Korea Centers for Disease Control and Prevention (2011E3300300, 2012E3301100, 2013E3301600, 2013E3301601, 2013E3301602, 2016E3300200, 2016E3300201, 2016E3300202, and 2019E320100), by a grant of the Korea Health Technology R&D Project through the Korea Health Industry Development Institute (KHIDI), funded by the Ministry of Health & Welfare, Republic of Korea (HR20C0021), and a grant (BCRI20076) of Chonnam National University Hospital Biomedical Research Institute.

## Conflict of Interest

The authors declare that the research was conducted in the absence of any commercial or financial relationships that could be construed as a potential conflict of interest.

## Publisher's Note

All claims expressed in this article are solely those of the authors and do not necessarily represent those of their affiliated organizations, or those of the publisher, the editors and the reviewers. Any product that may be evaluated in this article, or claim that may be made by its manufacturer, is not guaranteed or endorsed by the publisher.
