# Supplementary material for: Association of High Serum Adiponectin Level With Adverse Cardiovascular Outcomes and Progression of Coronary Artery Calcification in Patients With Pre-dialysis Chronic Kidney Disease
Source: Front Cardiovasc Med. 2022 Jan 13;8:789488. doi: 10.3389/fcvm.2021.789488 (PMC8792836; doi:10.3389/fcvm.2021.789488)
Supplement: Supplementary file 1 [file Data_Sheet_1.PDF]

Supplementary Material

Association of high serum adiponectin level with adverse cardiovascular outcomes and progression of coronary artery calcification in patients with pre-dialysis chronic kidney disease

Sang Heon Suh, M.D., Ph.D.[1], Tae Ryom Oh, M.D., Ph.D.[1], Hong Sang Choi, M.D., Ph.D.[1], Chang Seong Kim, M.D., Ph.D.[1], Joongyub Lee, M.D., Ph.D.[2], Yun Kyu Oh, M.D., Ph.D.[3], Ji Yong Jung, M.D., Ph.D[4], Kyu-Beck Lee, M.D., Ph.D[5], Kook-Hwan Oh, M.D., Ph.D.[3], Seong Kwon Ma, M.D., Ph.D.[1], Eun Hui Bae, M.D., Ph.D.[*, 1], and Soo Wan Kim, M.D., Ph.D.[*, 1], on behalf of the Korean Cohort Study for Outcomes in Patients With Chronic Kidney Disease (KNOW-CKD) Investigators

[1]Department of Internal Medicine, Chonnam National University Medical School, Gwangju, Republic of Korea
[2]Department of Prevention and Management, School of Medicine, Inha University, Incheon, Republic of Korea
[3]Department of Internal Medicine, Seoul National University College of Medicine, Seoul, Korea
[4]Division of Nephrology, Department of Internal Medicine, Gachon University of Gil Medical Center, Incheon, Republic of Korea
[5]Department of Internal Medicine, Kangbuk Samsung Hospital, Sungkyunkwan University School of Medicine, Seoul, Republic of Korea

Running title: Adiponectin and coronary calcification in CKD

Corresponding authors

*Eun Hui Bae, M.D., Ph.D., Department of Internal Medicine, Chonnam National University Medical School, 42 Jebongro, Gwangju 61469, Korea, Tel: +82-62-220-6503, Fax: +82-62-225-8578, Email: baedak76@gmail.com

*Soo Wan Kim, M.D., Ph.D., Department of Internal Medicine, Chonnam National University Medical School, 42 Jebongro, Gwangju 61469, Korea, Tel: +82-62-220-6271, Fax: +82-62-225-8578, Email: skimw@chonnam.ac.kr

Table of contents

Figure S1. Comparison of the probability for rapid progression of CAC during 4-year follow up by categorized CACS at the baseline
Figure S2. Restricted cubic spline of serum adiponectin on the risk of all-cause mortality
Figure S3. Restricted cubic spline of serum adiponectin on the risk of fatal and non-fatal CV events
Table S1. Binary logistic regression of serum adiponectin levels for progression of CAC in the subjects with eGFR < 90 mL/min./1.73m^2
Table S2. Cox regression analysis of serum adiponectin levels for fatal and non-fatal CV events in the subjects with eGFR < 90 mL/min./1.73m^2
Table S3. Cox regression analysis of serum adiponectin levels for clinical outcomes using a multiple imputation
Table S4. Binary logistic regression analysis of serum adiponectin levels for progression of CAC using a multiple imputation
Table S5. Binary logistic regression of serum adiponectin levels for progression of CAC in various subgroups

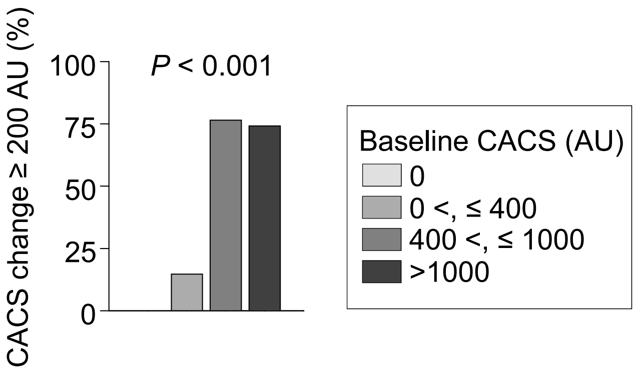

Figure S1. Comparison of the probability for rapid progression of CAC during 4-year follow up by categorized CACS at the baseline

Note: The probability for rapid progression of CAC during 4-year follow up were compared by categorized CACS at the baseline. *P* value by Chi square test. Abbreviations: AU, Agatston unit; CACS, coronary artery calcium score.

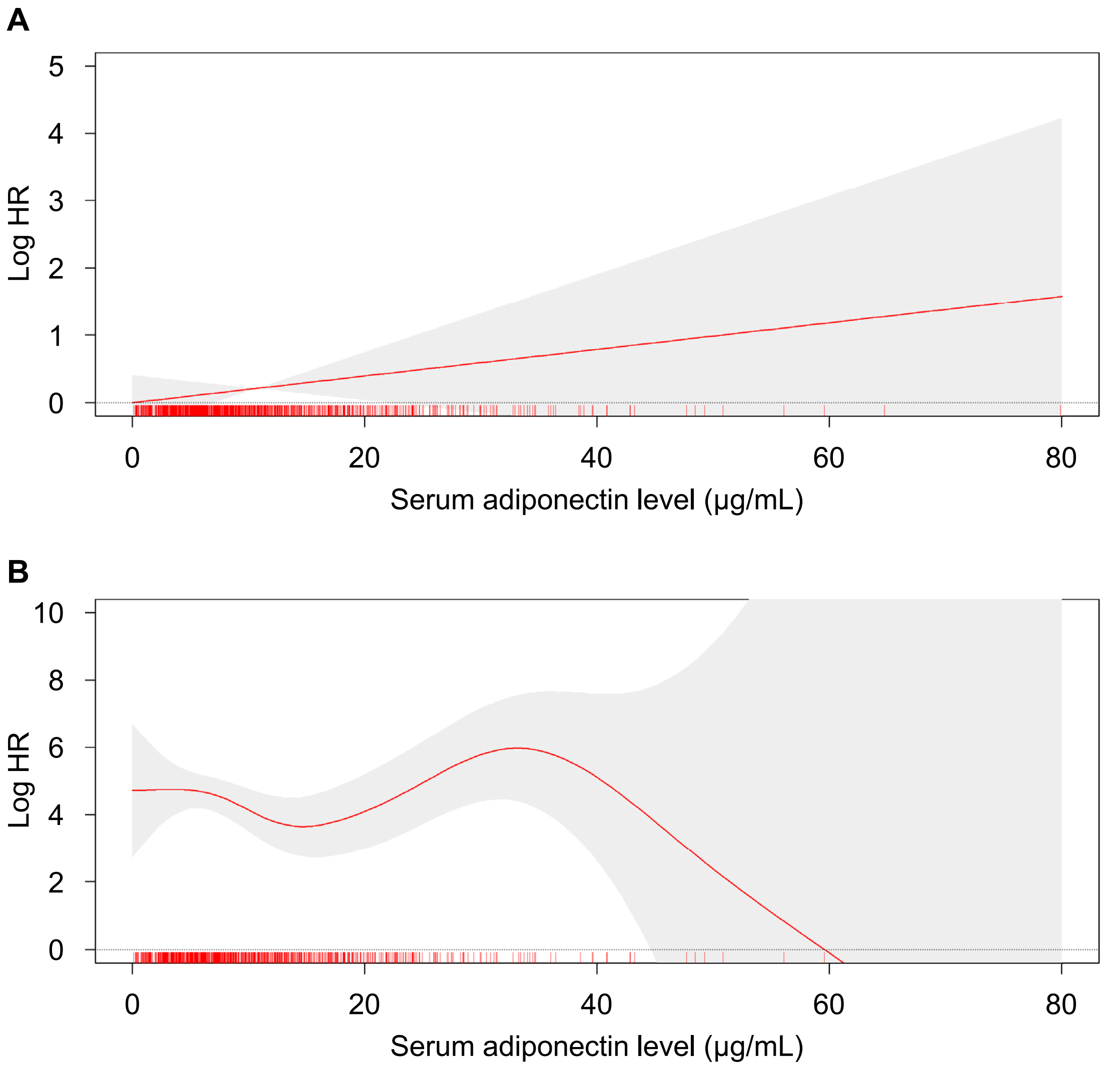

Figure S2. Restricted cubic spline of serum adiponectin on the risk of all-cause mortality

Note: Crude (A) and adjusted (B) HRs of serum adiponectin as a continuous variable for all-cause mortality are depicted. The model was adjusted for adjusted for age, gender, Charlson comorbidity index, history of DM, smoking history, BMI, WC, SBP, DBP, edications (ACEi/ARBs, statins, diuretics), hemoglobin, albumin, HDL-C, fasting glucose, hs-CRP, eGFR, 24-hour urine protein, and categorized CACS at the baseline. Abbreviations: HR, hazard ratio.

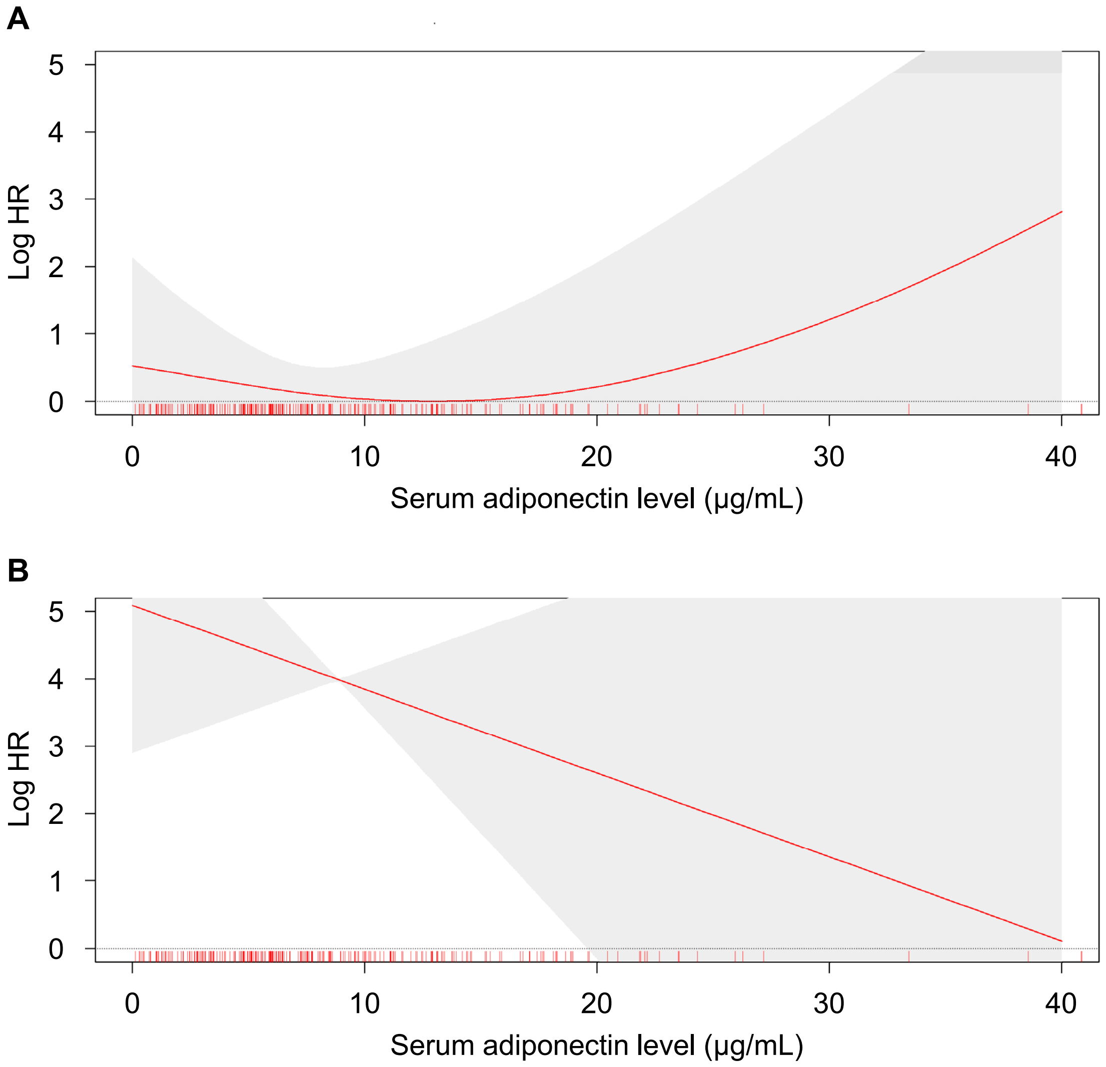

Figure S3. Restricted cubic spline of serum adiponectin on the risk of fatal and non-fatal CV events

Note: Adjusted HRs of serum adiponectin as a continuous variable for fatal and non-fatal CV events in the subjects with eGFR ≥ 72 mL/min./1.73m² (A) and ≥ 73 mL/min./1.73m² (B) are depicted. The model was adjusted for adjusted for age, gender, Charlson comorbidity index, history of DM, smoking history, BMI, WC, SBP, DBP, edications (ACEi/ARBs, statins, diuretics), hemoglobin, albumin, HDL-C, fasting glucose, hs-CRP, eGFR, 24-hour urine protein, and categorized CACS at the baseline. Abbreviations: HR, hazard ratio.

Table S1. Binary logistic regression of serum adiponectin levels for progression of CAC in the subjects with eGFR < 90 mL/min./1.73m^2

	Number of events (%)	Unadjusted OR (95%CIs)	*P* value	Adjusted OR (95%CIs)	*P* value
**Adiponectin, T1**	44 (24.8)	1.140 (0.661, 1.996)	0.638	1.159 (0.540, 2.486)	0.705
**Adiponectin, T2**	39 (22.5)	Reference		Reference	
**Adiponectin, T3**	38 (27.5)	1.287 (0.740, 2.240)	0.372	2.179 (1.038, 4.573)	0.040

Note: Event cases are given as number (percentage). Models were adjusted for age, gender, Charlson comorbidity index, history of DM, smoking history, BMI, WC, SBP, DBP, medications (ACEi/ARBs, statins, diuretics), hemoglobin, albumin, HDL-C, fasting glucose, hs-CRP, eGFR, 24-hour urine protein, and categorized CACS at the baseline. Abbreviations: CI, confidence interval; OR, odds ratio; T1, 1st tertile; T2, 2nd tertile; T3, 3rd tertile.

Table S2. Cox regression analysis of serum adiponectin levels for fatal and non-fatal CV events in the subjects with eGFR < 90 mL/min./1.73m^2

		Number of Events (%)	Unadjusted HR (95%CIs)	*P* value	Adjusted HR (95%CIs)	*P* value
**Fatal and non-fatal CV events**	**Adiponectin, T1**	19 (6.4)	1.292 (0.570, 2.928)	0.540	1.311 (0.553, 3.111)	0.539
	**Adiponectin, T2**	17 (5.3)	Reference		Reference	
	**Adiponectin, T3**	29 (8.9)	2.300 (1.137, 4.656)	0.021	3.096 (1.450, 6.609)	0.003
**All-cause mortality**	**Adiponectin, T1**	6 (2.0)	0.779 (0.255, 2.383)	0.662	0.815 (0.223, 2.981)	0.758
	**Adiponectin, T2**	9 (2.8)	Reference		Reference	
	**Adiponectin, T3**	8 (2.5)	0.694 (0.241, 2.001)		0.681 (0.213, 2.177)	0.517

Note: Models were adjusted for age, gender, Charlson comorbidity index, history of DM, smoking history, BMI, WC, SBP, DBP, medications (ACEi/ARBs, statins, diuretics), hemoglobin, albumin, HDL-C, fasting glucose, hs-CRP, eGFR, 24-hour urine protein, and categorized CACS at the baseline. Abbreviations: CI, confidence interval; HR, hazard ratio; T1, 1st tertile; T2, 2nd tertile; T3, 3rd tertile.

Table S3. Cox regression analysis of serum adiponectin levels for clinical outcomes using a multiple imputation

			Unadjusted HR (95%CIs)	*P* value	Adjusted HR (95%CIs)	*P* value
**All subjects**	**Fatal and non-fatal CV events**	**Adiponectin, T1**	1.221 (0.645, 2.312)	0.541	1.264 (0.647, 2.469)	0.493
		**Adiponectin, T2**	Reference		Reference	
		**Adiponectin, T3**	1.762 (0.985, 3.154)	0.056	2.099 (1.127, 3.908)	0.019
	**All-cause mortality**	**Adiponectin, T1**	0.810 (0.288, 2.276)	0.689	1.071 (0.350, 3.275)	0.904
		**Adiponectin, T2**	Reference		Reference	
		**Adiponectin, T3**	1.030 (0.409, 2.595)	0.950	0.958 (0.349, 2.630)	0.933
**eGFR < 90 mL/min./1.73m^2**	**Fatal and non-fatal CV events**	**Adiponectin, T1**	1.104 (0.556, 2.189)	0.778	1.175 (0.573, 2.409)	0.659
		**Adiponectin, T2**	Reference		Reference	
		**Adiponectin, T3**	1.628 (0.890, 2.979)	0.114	1.950 (1.019, 3.733)	0.044
	**All-cause mortality**	**Adiponectin, T1**	0.855 (0.304, 2.403)	0.766	1.185 (0.380, 3.693)	0.770
		**Adiponectin, T2**	Reference		Reference	
		**Adiponectin, T3**	0.877 (0.328, 2.273)	0.787	0.874 (0.307, 2.492)	0.802

Note: Models were adjusted for age, gender, Charlson comorbidity index, history of DM, smoking history, BMI, WC, SBP, DBP, medications (ACEi/ARBs, statins, diuretics), hemoglobin, albumin, HDL-C, fasting glucose, hs-CRP, eGFR, 24-hour urine protein, and categorized CACS at the baseline. Abbreviations: CI, confidence interval; HR, hazard ratio; T1, 1st tertile; T2, 2nd tertile; T3, 3rd tertile.

Table S4. Binary logistic regression analysis of serum adiponectin levels for progression of CAC using a multiple imputation

		Unadjusted HR (95%CIs)	*P* value	Adjusted HR (95%CIs)	*P* value
**All subjects**	**Adiponectin, T1**	1.056 (0.650, 1.716)	0.826	1.015 (0.527, 1.953)	0.965
	**Adiponectin, T2**	Reference		Reference	
	**Adiponectin, T3**	1.331 (0.806, 2.199)	0.264	1.793 (0.928, 3.465)	0.082
**eGFR < 90 mL/min./1.73m^2**	**Adiponectin, T1**	1.064 (0.635, 1.783)	0.815	1.093 (0.538, 2.220)	0.806
	**Adiponectin, T2**	Reference		Reference	
	**Adiponectin, T3**	1.383 (0.820, 2.331)	0.224	2.055 (1.026, 4.117)	0.042

Note: Models were adjusted for age, gender, Charlson comorbidity index, history of DM, smoking history, BMI, WC, SBP, DBP, medications (ACEi/ARBs, statins, diuretics), hemoglobin, albumin, HDL-C, fasting glucose, hs-CRP, eGFR, 24-hour urine protein, and categorized CACS at the baseline. Abbreviations: CI, confidence interval; HR, hazard ratio; T1, 1st tertile; T2, 2nd tertile; T3, 3rd tertile.

Table S5. Binary logistic regression of serum adiponectin levels for progression of CAC in various subgroups

		Number of events (%)	Unadjusted OR (95%CIs)	*P* for interaction	Adjusted OR (95%CIs)	*P* for interaction
**Age < 60 years**	**Adiponectin, T1**	22 (7.9)	1.759 (0.826, 3.747)	0.534	2.539 (0.725, 8.889)	0.047
	**Adiponectin, T2**	12 (4.7)	Reference		Reference	
	**Adiponectin, T3**	13 (5.0)	0.964 (0.408, 2.277)		1.550 (0.354, 6.788)	
**Age ≥ 60 years**	**Adiponectin, T1**	22 (22.4)	0.848 (0.418, 1.719)		0.473 (0.163, 1.372)	
	**Adiponectin, T2**	29 (23.0)	Reference		Reference	
	**Adiponectin, T3**	27 (23.5)	1.005 (0.528, 1.911)		2.395 (0.933, 6.149)	
**Male**	**Adiponectin, T1**	37 (13.1)	0.795 (0.459, 1.377)	0.444	0.832 (0.376, 1.838)	0.326
	**Adiponectin, T2**	29 (13.6)	Reference		Reference	
	**Adiponectin, T3**	28 (17.2)	1.102 (0.613, 1.982)		2.022 (0.835, 4.899)	
**Female**	**Adiponectin, T1**	7 (7.6)	1.292 (0.431, 3.871)		4.036 (0.356, 45.744)	
	**Adiponectin, T2**	12 (7.3)	Reference		Reference	
	**Adiponectin, T3**	12 (5.7)	1.013 (0.388, 2.644)		9.494 (0.806, 111.789)	
**DM (-)**	**Adiponectin, T1**	11 (4.3)	0.792 (0.355, 1.768)	0.487	0.418 (0.120, 1.458)	0.092
	**Adiponectin, T2**	15 (5.2)	Reference		Reference	
	**Adiponectin, T3**	12 (4.1)	0.609 (0.268, 1.387)		1.755 (0.520, 6.051)	
**DM (+)**	**Adiponectin, T1**	33 (28.0)	0.968 (0.499, 1.879)		1.966 (0.717, 5.391)	
	**Adiponectin, T2**	26 (28.0)	Reference		Reference	
	**Adiponectin, T3**	28 (35.4)	1.614 (0.805, 3.238)		2.715 (0.919, 8.024)	
**eGFR ≥ 60 mL/min./1.73m^2**	**Adiponectin, T1**	19 (9.9)	1.170 (0.526, 2.602)	0.190	0.178 (0.030, 1.077)	0.192
	**Adiponectin, T2**	11 (7.6)	Reference		Reference	
	**Adiponectin, T3**	5 (4.0)	0.402 (0.124, 1.303)		0.192 (0.021, 1.729)	
**eGFR < 60 mL/min./1.73m^2**	**Adiponectin, T1**	25 (13.7)	1.040 (0.558, 1.940)		1.433 (0.561, 3.663)	
	**Adiponectin, T2**	30 (12.8)	Reference		Reference	
	**Adiponectin, T3**	35 (14.1)	1.127 (0.642, 1.978)		3.504 (1.492, 8.231)	
**Spot urine ACR < 300 mg/g**	**Adiponectin, T1**	20 (9.0)	1.089 (0.507, 2.340)	0.290	0.628 (0.185, 2.130)	0.339
	**Adiponectin, T2**	14 (6.9)	Reference		Reference	
	**Adiponectin, T3**	19 (9.7)	1.335 (0.626, 2.849)		2.060 (0.624, 6.798)	
**Spot urine ACR ≥ 300 mg/g**	**Adiponectin, T1**	24 (16.3)	1.090 (0.573, 2.073)		0.709 (0.232, 2.165)	
	**Adiponectin, T2**	15 (15.5)	Reference		Reference	
	**Adiponectin, T3**	19 (11.2)	0.692 (0.356, 1.345)		1.151 (0.392, 3.383)	

Note: Models were adjusted for age, gender, Charlson comorbidity index, history of DM, smoking history, BMI, WC, SBP, DBP, medications (ACEi/ARBs, statins, diuretics), hemoglobin, albumin, HDL-C, fasting glucose, hs-CRP, eGFR, 24-hour urine protein, and categorized CACS at the baseline. Abbreviations: ACR, albumin-to-creatinine ratio; CI, confidence interval; DM, diabetes mellitus; eGFR, estimated glomerular filtration rate; OR, odds ratio; T1, 1st tertile; T2, 2nd tertile; T3, 3rd tertile.
